# Molecular Evidence of a Broad Range of Pathogenic Bacteria in *Ctenocephalides* spp.: Should We Re-Examine the Role of Fleas in the Transmission of Pathogens?

**DOI:** 10.3390/tropicalmed6010037

**Published:** 2021-03-17

**Authors:** Georgios Dougas, Athanassios Tsakris, Stavroula Beleri, Eleni Patsoula, Maria Linou, Charalambos Billinis, Joseph Papaparaskevas

**Affiliations:** 1 Department of Microbiology, Medical School, National and Kapodistrian University of Athens, 115 27 Athens, Greece; atsakris@med.uoa.gr (A.T.); ipapapar@med.uoa.gr (J.P.); 2 Department of Public Health Policy, School of Public Health, University of West Attica, 11521 Athens, Greece; smpeleri@uniwa.gr (S.B.); epatsoula@uniwa.gr (E.P.); 3 Hellenic Pasteur Institute, 11521 Athens, Greece; linoum@pasteur.gr; 4 Faculty of Veterinary Science, University of Thessaly, 43100 Karditsa, Greece; cbillinis@gmail.com; 5 Faculty of Public and Integrated Health, University of Thessaly, 43100 Karditsa, Greece

**Keywords:** metagenomics, disease transmission, infectious, *Ctenocephalides*, flea infestations, bacteremia, *Klebsiella pneumoniae*, *Enterococcus faecalis*, *Pseudomonas aeruginosa*, *Legionella*

## Abstract

The internal microbiome of common cat and dog fleas was studied for DNA evidence of pathogenic bacteria. Fleas were grouped in pools by parasitized animal. DNA was extracted and investigated with 16S metagenomics for medically relevant (MR) bacteria, based on the definitions of the International Statistical Classification of Diseases and Related Health Problems (WHO). The MR bacterial species totaled 40, were found in 60% of flea-pools (N = 100), and included *Acinetobacter* *baumannii*, *Bacteroides* *fragilis*, *Clostridium* *perfringens*, *Enterococcus* *faecalis*, *E*. *mundtii*, *Fusobacterium* *nucleatum*, *Haemophilus* *aegyptius*, *Kingella* *kingae*, *Klebsiella* *pneumoniae*, *Leptotrichia* *buccalis*, *L*. *hofstadii*, *Moraxella* *lacunata*, *Pasteurella* *multocida*, *Propionibacterium* *acnes*, *P*. *propionicum*, *Proteus* *mirabilis*, *Pseudomonas* *aeruginosa*, *Rickettsia* *australis*, *R*. *hoogstraalii*, *Salmonella* *enterica*, and various *Bartonella*, *Staphylococcus*, and *Streptococcus* species. *B*. *henselae* (*p* = 0.004) and *B*. *clarridgeiae* (*p* = 0.006) occurred more frequently in fleas from cats, whereas *Rickettsia* *hoogstraalii* (*p* = 0.031) and *Propionibacterium* *acnes* (*p* = 0.029) had a preference in fleas from stray animals. Most of the discovered MR species can form biofilm, and human exposure may theoretically occur through the flea-host interface. The fitness of these pathogenic bacteria to cause infection and the potential role of fleas in the transmission of a broad range of diseases should be further investigated.

## 1. Introduction

**Figure 1:**
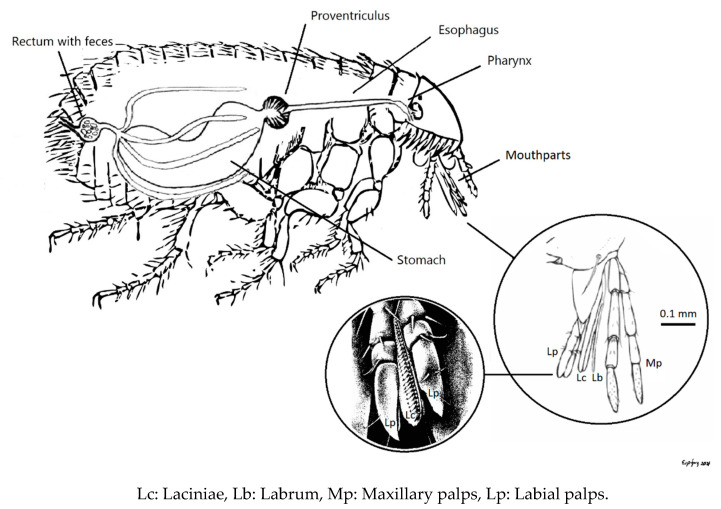
Flea (*Ctenocephalides felis*) digestive system, schematic representation. Mouthparts bear dents and cilia-like projections. Laciniae have serrated, saw-like, sharp surfaces for penetration of the skin.

Fleas are obligate blood-sucking ectoparasites, with world-wide distribution. *Ctenocephalides felis* and *C*. *canis* commonly parasitize cats and dogs and represent the most abundant flea species [1]. Moreover, these flea species may also feed on other mammals, including humans [2]. During feeding, the skin of the host is penetrated by labrum, a sclerite stylet, and the laciniae, a pair of serrated saw-like mandibles. Saliva, with anticoagulant properties, is injected in the host dermis through two canals running across each of the laciniae. Subsequently, blood of the host is drawn and passed to esophagus, proventriculus, and stomach, where digestion takes place (Figure 1). Fleas deposit feces on the host which may fall off and spill over to the environment [3].

The *Ctenocephalides* spp. fleas are efficient vectors of *Rickettsia felis* [4,5] and various *Bartonella* species [6,7]. However, studies based on the analysis of the 16S rDNA gene, revealed a wide diversity of bacterial species in the common flea microbiome [8,9,10]. The automation of the technique, using commercially available high-throughput sequencing platforms, permit the simultaneous investigation of a vast range of bacterial genera and species in environmental specimens. These molecular diagnostic tools cancel the need for conventional culture and allow the identification of fastidious, even non-culturable, bacteria.

In that respect, the investigation of the bacterial flora of the gastrointestinal tract of the flea may provide new insights on pathogen transmission routes. 

In the present study, we investigated the microbiome of fleas collected from pet animals in Attica, Greece, using 16S metagenomics, with a special focus on pathogenic bacteria. Furthermore, we examined the flea-human interface for possible transmission pathways and laid down a hypothesis of a potential role of fleas as vectors of a broad range of infectious agents.

## 2. Materials and Methods

Fleas were collected by combing or forceps, from dogs and cats presented in collaborating veterinary clinics, during the 2016−2017 period, in the region of Attica, Greece. 

The fleas were identified using morphological criteria [11] and grouped in flea-pools stratified by individual animal-host, and insect genus, species, and sex. A total of 100 representative flea-pools from an equal number of animals, comprising only female insects, were selected for analysis. 

The exoskeleton of the fleas was cleaned from impurities and external flora according to a previously described protocol [12]. The insects of each flea-pool were homogenized by thrusting with a sterile pestle for at least 90 s, or for as long as was required until no further macroscopical disruption was possible. The DNA was extracted with QIAamp DNA Mini Kit (Qiagen, Hilden, Germany) according to manufacturer’s instructions and the tissue protocol.

The samples were investigated with 16S Next Generation Sequencing (Abbrv.: 16S) with the Ion 16S Metagenomics kit in a PGM Ion Torrent platform (Thermo Fisher Scientific, Waltham, MA, USA). The amplicon sequence reads were clustered in Operational Taxonomic Units, and the cut-offs for identification of genus and species level were 97% and 99%, respectively. The metagenomics data were compared with the 16S rRNA sequences in MicroSEQ v2013.1 and GreenGenes v13.5 curated databases and analyzed by QIIME ver. 2.

The designation of medically relevant (Abbr.: MR) bacteria was based on the International Statistical Classification of Diseases and Related Health Problems of WHO (ICD-11, 09/2020 version). Specifically, MR bacterial genera and species were limited to the ones described as causative agents of infectious diseases in the sections (i) X Extension Codes/Aetiology/Infectious agents and (ii) Mortality and Morbidity Statistics Special tabulation list of infectious agents/ Infectious diseases by infectious agent/Infections due to Bacteria.

The areas of residence of the host-animals were classified to predominantly urban, intermediate, and predominantly rural, according to Eurostat regional nomenclature [13]. 

All experiments were performed in a molecular microbiology-dedicated section of the lab premises, where no handling of cultures, clinical specimens, nor isolates was performed. Negative control (dH_2_O) was used in all experiments. 

Statistical analysis was accomplished with IBM SPSS Statistics for Windows, Version 20.0 (IBM Corp., Armonk, NY, USA), and a value of *p* < 0.05 was considered significant.

## 3. Results

### 3.1. Flea Samples

The flea-pools were collected from 67 cats and 33 dogs. The mean age of host animals was 27.8 months (95% CI: 20.1−35.5), with a predominance of stray females (female to male ratio 1.7:1, stray to owned ratio 2.4:1). All animals resided in the Attica region, which is a predominantly urban area. The great majority of the samples were populated by *Ctenocephalides felis* (n = 96), and the rest by *C*. *canis* (n = 2) and *Pulex irritans* (n = 2), with a median of 2 insects per sample (IQR:1–4). The flea-pools collected from cats (median = 3; IQR:1–5) had significantly more fleas (U = 651.5, *p* = 0.001) compared with those from dogs (median = 1; IQR:1–2). The retrieved flea species by host animal species, gender, age, and status are summarized in Table 1.

### 3.2. Flea Internal Microbiome

**Figure 2:**
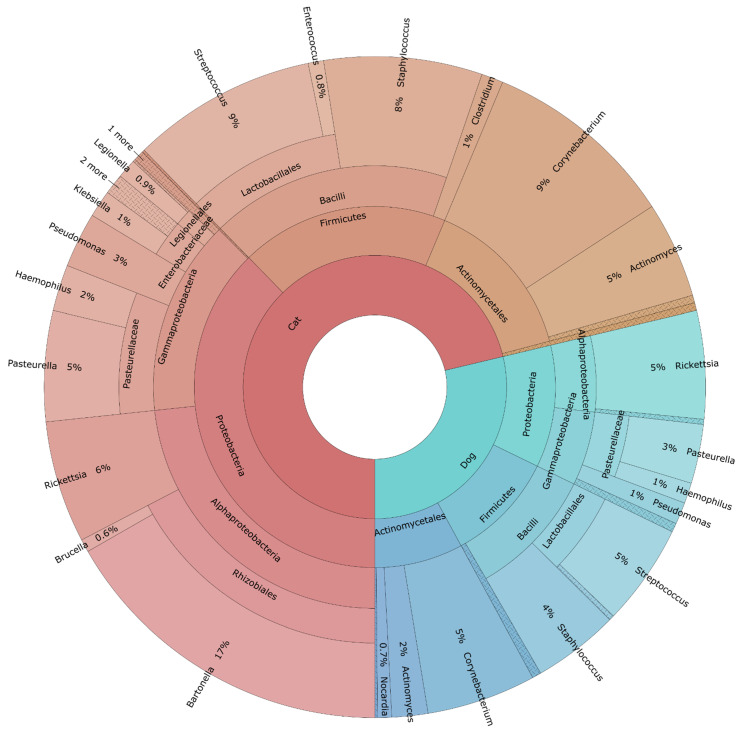
Krona chart of bacterial genera identified with 16S Metagenomics in flea-pools (N = 100) from cats and dogs in Attica, Greece.

A total of 18 phyla, 318 genera, and 468 species of bacteria were detected (see Supplementary Material, Table S1). Proteobacteria was the most abundant phylum recovered in 100% of flea-pools, followed by Firmicutes, Actinobacteria, Bacteroides, Tenericutes, Fusobacteria, Nitrospirae, Acidobacteria, Cyanobacteria, Spirochaetes, Acidobacteria, Fusobacteria, Deinococcus-Thermus, Nitrospirae, Planctomycetes, Chlamydiae, Synergistetes, Deferribacteres, and Gemmatinomadetes. The 10 most abundant genera were *Wolbachia*, *Pasteurella*, *Acinetobacter*, *Diaphorobacter*, *Streptococcus*, *Staphylococcus*, *Bartonella*, *Lactobacillus*, *Gallibacterium*, and *Corynebacterium* spp. (Figure 2). 

### 3.3. Medically Relevant Bacterial Genera

**Figure 3:**
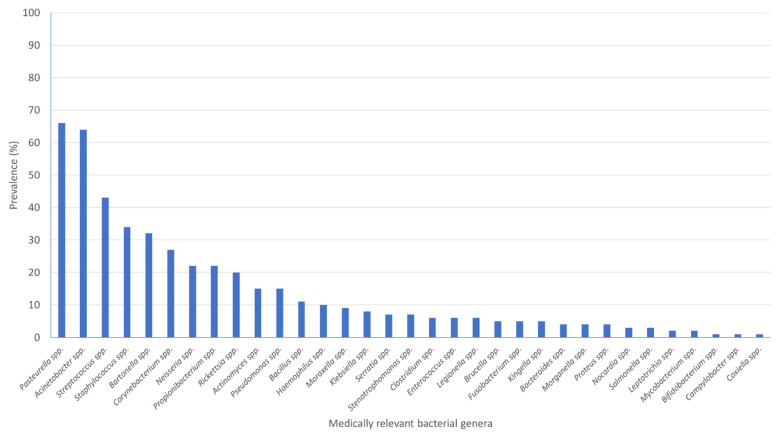
Prevalence of medically relevant (MR) bacterial genera according to the International Statistical Classification of Diseases and Related Health Problems by WHO (ICD-11, 2020 version) in flea-pools (N = 100) collected from cats and dogs in Attica, Greece.

A total of 33 MR genera were identified in 96 flea-pools, with a mean of 4.70 MR genera (95% CI: 3.74, 5.66) per flea-pool (Figure 3). 

Among the 1418 detections instances of MR genera by 16S, only in 627 (44.2%) was a species identified. The 16S mapped valid reads were fully attributed to species for five MR genera (*Propionibacterium*, *Proteus*, *Salmonella*, *Leptotrichia*, and *Morganella*), and only partially for 23 MR genera, whereas no species was retrieved for five MR genera (*Brucella*, *Bifidobacterium*, *Campylobacter*, *Coxiella*, and *Mycobacterium*) (see Supplementary material, Figure S1). 

### 3.4. Medically Relevant Bacterial Species

**Figure 4:**
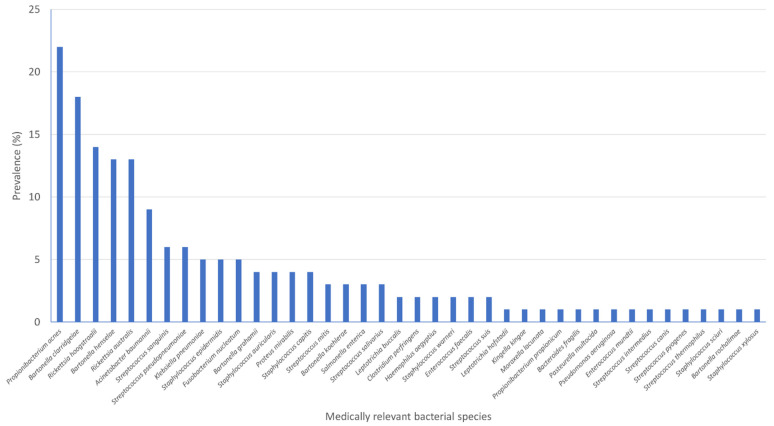
Prevalence of medically relevant (MR) bacterial species according to the International Statistical Classification of Diseases and Related Health Problems by WHO (ICD-11, 2020 version) in flea-pools (N = 100) collected from cats and dogs in Attica, Greece.

MR bacterial species were found in 60 flea-pools with a mean of 1.71 MR species (95% CI: 1.14, 2.28) per sample (N = 100) (Figure 4). In total, 40 MR species were identified (Table 2). 

The total count of MR species did not differ between specimens collected from cats and dogs (*p* = 0.276) and was not affected by the gender (*p* = 0.998), the stray status (*p* = 0.982), the age of the animal (*p* = 0.759), nor the number of insects in the flea-pool (*p* = 0.463). 

*Bartonella clarridgeiae* (X^2^(1) = 7.478, *p* = 0.006) and *B. henselae* (Fisher’s exact test, *p* = 0.004) were strongly associated with fleas collected from cats. *Rickettsia hoogstraalii* had a preference for stray animals (X^2^(2) = 6.977, *p* = 0.031) and, similarly, *Propionibacterium acnes* (X^2^(2) = 7.051, *p* = 0.029) (see Supplementary material, Table S2).

## 4. Discussion

The present study was designed to investigate the microbiome of fleas parasitizing cats and dogs for potential pathogens using a high throughput 16S next generation sequencing. To our knowledge, this is the first systematic survey of flea samples with the aim to explore a broad range of bacteria. 

The flea-associated bacteria *Bartonella* and *Rickettsia* spp. were detected in 32% and 20% of the flea-pools, respectively. *B*. *clarridgeiae* was the dominant species for the *Bartonella* genus, followed by *B*. *henselae*, *B*. *grahamii*, *B. koehlerae*, *B*. *rattaustraliani*, and *B*. *rochalimae*. *Bartonella* species were strongly associated with fleas from cat-hosts. Among *Rickettsia* species, *R*. *hoogstraalii* had the highest occurrence rate, followed by *R*. *australis*. However, *R*. *hoogstraalii*, *R*. *australis*, *R*. *felis*, *R*. *senegalensis*, and *R*. *akari* are phylogenetically close, forming the *R*. *felis* group clade, and cannot be distinguished by 16S [14,15]. The findings of the study regarding *Bartonella* and *Rickettsia* bacteria concur with similar reports [6,16,17,18].

However, the present study, apart from *Bartonella* and *Rickettsia*, yielded molecular evidence for 38 additional MR bacterial species in the microbiome of fleas collected from cats and dogs. Noteworthily, MR bacteria occurred at a significant percentage of flea-pools but also of individual insects, regardless of host species, sex, age, or stray status. 

Even though the *Ctenocephalides* fleas have been associated with specific pathogens, i.e., *Bartonella* and *Rickettsia*, human infection with the other bacteria would also occur if infectiousness was preserved and exposure was possible through the flea-human interface. Our study provided only DNA evidence, without assessing the vitality and fitness for infection of the MR bacteria. However, the majority of the MR species detected in this study are reported as capable of forming biofilm (e.g., Streptococci, Staphylococci, *Proteus*, *Pseudomonas*, *Stenotrophomonas*, etc.). Biofilm formation is a common strategy of microorganisms to withstand environmental adversities [19]. Bacterial colonies surrounded by biofilm have greater chance of survival either attached on flea anatomical parts or within flea-feces. The laciniae skin-piercing instrument and the surrounding maxillary and labial palps of the flea mouthparts have irregular surfaces with serration, dents, and cilia, niches promoting bacterial colonization (Figure 1). Inoculation of detached bacterial cells to human tissues might be possible during blood feeding. A non-biological transmission pathway via contaminated flea mouthparts has been suggested for the flea-borne pathogens *Y. pestis* and *R. felis* [20,21]. A similar direct ‘dirty-needle-like’ mechanical transfer of pathogens would also apply when it comes to human exposure to MR bacteria.

Well-established transmission routes of known flea-borne pathogens from fleas to humans include the inoculation of the skin with flea saliva or feces. Saliva inoculation occurs during feeding on the host (*R*. *felis*) [4]. The inoculation with flea-feces is mediated by cat claws (*Bartonella* spp.) [7]. A human may also rub the pathogen into the skin during scratching of the pruritic flea-bite lesion (*R*. *typhi*) [22]. These transmission pathways could be theoretically employed by other bacteria; however, we are not aware of studies on flea mouthparts, saliva, or feces for potential pathogenic flora. 

Of interest is the bacteremia of unknown origin (BUO), a community-acquired blood infection which cannot be attributed to a specific infectious locus, and the causative bacteria can be retrieved only from the blood of the patient. Reports estimate 40 to 154 cases per 100,000 annually [23] with a mortality rate higher than that of the nosocomial-acquired bacteremia [24]. Among the MR bacteria detected in the flea-pools of our study, the following are described as causative agents of BUO: *B. fragilis*, *K. pneumoniae*, *P. mirabilis*, *P. aeruginosa*, *E. faecalis*, and *F. nucleatum* [24,25]. However, bacteria not satisfying the ICD-11 criterion, but reported as BUO causative agents, were also detected: *Streptococcus equi*, *Elizabethkingia miricola*, *Gemella haemolysans*, *Parvimonas micra*, *Eubacterium hadrum*, *E*. *saphenum*, *E*. *ramulus*, *E*. *brachy*, and *E*. *hallii* [25,26,27,28,29]. A study in a rural area of Democratic Republic of Congo provided evidence of human blood in approximately 10% of fleas caught indoors [30]. We are not aware of similar studies in urban settings; however, we speculate that encounters of fleas with humans, even though scarcer compared with the ones with animals, cumulatively, may represent a significant count of total exposures per human lifetime. We suggest that an epidemiological link of BUO with fleas should be investigated as it could possibly provide an explanation for a fraction of cases.

Several MR bacteria of our study are foodborne disease agents, such as *C. perfringens*, *S. enterica*, and *Campylobacter* spp. The vehicle of infection in about a third of foodborne outbreaks remains unidentified [31]. Sporadic cases represent a significant proportion of the foodborne cases, and, in total count, outnumber the outbreak-related ones; however, they usually remain uninvestigated [32]. We postulate that accidental ingestion of flea-feces, or even whole insects, could possibly explain a percentage of sporadic infections. We are not aware of studies assessing the environmental spill-over of flea fecal material or the extent of human exposure. A systematic study of the dynamics of flea-mediated transmission of foodborne pathogens may provide additional epidemiological insight for the related diseases.

Furthermore, the list of pathogenic bacteria of the flea microbiome might be longer since the 16S resolution failed to provide species identification in a significant proportion of MR genus detection instances, e.g., *Brucella* spp. was detected in five samples without species determination, although these samples were later proven positive for *B. melitensis* using genus and species-specific PCR [33]. Similarly, 16S sequences of *Legionella* genus were identified in six samples but species remained undetermined in all cases. 

Both male and female fleas require blood meals for mating but females are reported as marginally more aggressive in blood consumption compared to males (*p* > 0.05) [34,35]. The authors also assumed that the egg producing females might be in higher demand for blood. This study included only female insects based on the postulation that intensified blood sucking may entail more risk for pathogen transmission. Nevertheless, focusing only in females might have introduced bias, and male fleas should be included in future studies.

This is the first report for evidence of a variety of pathogenic bacteria in the microbiome of common pet fleas. However, it should be emphasized that this study has only focused in DNA evidence provided by 16S without further validation of the results with species-specific confirmatory molecular techniques. On top of that, the limitations of the 16S in correctly discriminating species within certain genera are well known (e.g., *Bacillus* and/or *Streptococcus* spp.) [36,37]. 

Furthermore, and importantly, the study did not assess the viability and fitness for infection of the identified bacteria and did not specifically examine the microbiome of the flea mouthparts, saliva, and feces, more likely associated with potential human exposure. 

## 5. Conclusions 

A broad range of pathogenic bacteria were found to be widely distributed in common fleas parasitizing cats and dogs, as evidenced by a 16S metagenomics platform. Many aspects of the flea-host interface present a theoretical possibility for human exposure to pathogenic bacteria, other than the established flea-borne ones. In that respect, more research is needed in order to explore a potential roadmap of human infection involving fleas.

## Figures and Tables

**Table 1:** Species, age-group, gender, and ownership status of host animals by parasitizing flea species (N = 100).

Host Species	Host Age-Group	Host Gender	Host Status	Flea Species	Flea-Pools
Cat	0−1 years	Female	Stray	*C. felis*	24
			Owned	*C. felis*	2
		Male	Stray	*C. felis*	15
			Unknown	*C. felis*	1
	1−5 years	Female	Stray	*C. felis*	9
			Owned	*C. felis*	1
		Male	Stray	*C. felis*	5
			Owned	*C. felis*	1
	>5 years	Female	Stray	*C. felis*	2
			Owned	*C. felis*	4
		Male	Stray	*C. felis*	1
	Unknown	Female	Stray	*C. felis*	2
Dog	0−1 years	Female	Stray	*C. felis*	2
			Owned	*C. felis*	1
				*P. irritans*	1
		Male	Stray	*C. felis*	3
				*C. canis*	1
			Owned	*C. felis*	4
				*C. canis*	1
		Unknown	Stray	*P. irritans*	1
	1−5 years	Female	Stray	*C. felis*	3
			Owned	*C. felis*	7
		Male	Stray	*C. felis*	2
			Owned	*C. felis*	2
	>5 years	Female	Owned	*C. felis*	4
		Male	Owned	*C. felis*	1

**Table 2:** Medically relevant (MR) bacteria associated with infectious diseases, as described in International Statistical Classification of Diseases and Related Health Problems by WHO (ICD-11, 2020), detected by 16S Metagenomics in the internal microbiome of fleas (n = 100 flea-pools) collected from cats and dogs in Attica, Greece.

MR Bacterial Genera	MR Bacterial Species	No of Flea-Pools	MR Bacterial Genera	MR Bacterial Species	No of Flea-Pools
*Acinetobacter*	*baumannii*	9	*Mycobacterium*	Species unidentified	2
	Species unidentified	58	*Neisseria*	Species unidentified	15
*Actinomyces*	Species unidentified	12	*Nocardia*	Species unidentified	3
*Bacillus*	Species unidentified	10	*Pasteurella*	*multocida*	1
*Bacteroides*	*fragilis*	1		Species unidentified	65
	Species unidentified	1	*Propionibacterium*	*acnes*	22
*Bartonella*	*clarridgeiae*	18		*propionicum*	1
	*grahamii*	4		Species unidentified	0
	*henselae*	13	*Proteus*	*mirabilis*	4
	*koehlerae*	3		Species unidentified	0
	*rochalimae*	1	*Pseudomonas*	*aeruginosa*	1
	Species unidentified	35		Species unidentified	15
*Bifidobacterium*	Species unidentified	1	*Rickettsia*	*australis*	13
*Brucella*	Species unidentified	5		*hoogstraalii*	14
*Campylobacter*	Species unidentified	1		Species unidentified	20
*Clostridium*	*perfringens*	2	*Salmonella*	*enterica*	3
	Species unidentified	6		Species unidentified	0
*Corynebacterium*	Species unidentified	24	*Serratia*	Species unidentified	7
*Coxiella*	Species unidentified	1	*Staphylococcus*	*auricularis*	4
*Enterococcus*	*faecalis*	2		*capitis*	4
	*mundtii*	1		*epidermidis*	5
	Species unidentified	4		*sciuri*	1
*Fusobacterium*	*nucleatum*	5		*warneri*	2
	Species unidentified	1		*xylosus*	1
*Haemophilus*	*aegyptius*	2		Species unidentified	31
	Species unidentified	6	*Stenotrophomonas*	Species unidentified	3
*Kingella*	*kingae*	1	*Streptococcus*	*canis*	1
	Species unidentified	1		*intermedius*	1
*Klebsiella*	*pneumoniae*	5		*mitis*	3
	Species unidentified	3		*pseudopneumoniae*	6
*Legionella*	Species unidentified	6		*pyogenes*	1
*Leptotrichia*	*buccalis*	2		*salivarius*	3
	*hofstadii*	1		*sanguinis*	6
	Species unidentified	0		*suis*	2
*Moraxella*	*lacunata*	1		*thermophilus*	1
	Species unidentified	5		Species unidentified	43
*Morganella*	Species unidentified	0			

## Data Availability

The data presented in this study are available in Supplementary Materials Tables S1 and S2, Figure S1.

## Supplementary Materials

The following are available online at https://www.mdpi.com/2414-6366/6/1/37/s1, Table S1: 16S Metagenomics internal microbiome of flea-pools collected from pet animals in Greece (N = 100), Table S2: Animal data, total fleas and medically relevant (MR) bacterial species, per flea-pool in flea-pools collected from pet animals in Greece (N = 100), Figure S1: Percentage of bacterial species identification in detections of medically relevant (MR) genera detections by 16S Metagenomics, in flea-pools collected from pet animals in Greece (N = 100).
