# Supplementary figures and images for: Molecular Evidence of a Broad Range of Pathogenic Bacteria in Ctenocephalides spp.: Should We Re-Examine the Role of Fleas in the Transmission of Pathogens?
Source: Trop Med Infect Dis. 2021 Mar 17;6(1):37. doi: 10.3390/tropicalmed6010037 (PMC8005979; doi:10.3390/tropicalmed6010037)

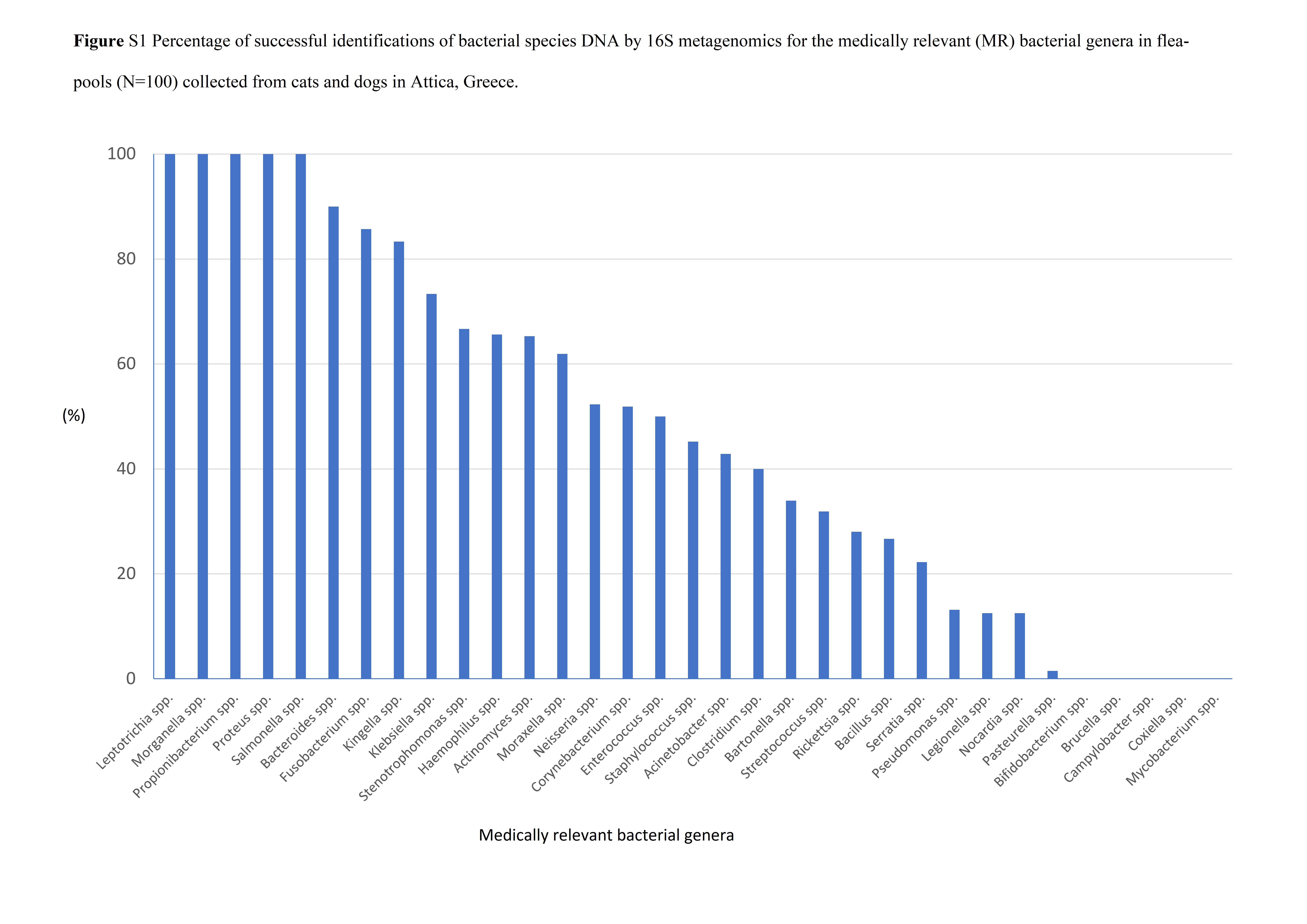

Supplement: Supplementary file 1 [file tropicalmed-06-00037-s001.zip › Supplementary files/Figure S1.tif]
